# Facile synthesis, microstructure and photophysical properties of core-shell nanostructured (SiCN)/BN nanocomposites

**DOI:** 10.1038/srep39866

**Published:** 2017-01-13

**Authors:** Qian Zhang, Dechang Jia, Zhihua Yang, Delong Cai, Richard M. Laine, Qian Li, Yu Zhou

**Affiliations:** 1 Institute for Advanced Ceramics, School of Materials Science and Engineering, Harbin Institute of Technology, Harbin 150001, PR China; 2 College of Engineering Materials Science and Engineering, University of Michigan, Michigan, United State

## Abstract

Increasing structural complexity at nanoscale can permit superior control over photophysical properties in the precursor-derived semiconductors. We demonstrate here the synthesis of silicon carbonitride (SiCN)/boron nitride (BN) nanocomposites via a polymer precursor route wherein the cobalt polyamine complexes used as the catalyst, exhibiting novel composite structures and photophysical properties. High Resolution Transmission Electron Microscopy (HRTEM) analysis shows that the diameters of SiCN−BN core−shell nanocomposites and BN shells are 50‒400 nm and 5‒25 nm, respectively. BN nanosheets (BNNSs) are also observed with an average sheet size of 5‒15 nm. The photophysical properties of these nanocomposites are characterized using the UV-Vis and photoluminescence (PL) analyses. The as-produced composites have emission behavior including an emission lifetime of 2.5 ns (±20 ps) longer observed in BN doped SiCN than that seen for SiC nanoparticles. Our results suggest that the SiCN/BN nanocomposites act as semiconductor displaying superior width photoluminescence at wavelengths spanning the visible to near-infrared (NIR) spectral range (400‒700 nm), owing to the heterojunction of the interface between the SiC(N) nanowire core and the BN nanosheet shell.

Material fusions in heterostructures with novel properties different from those of the constituents have become one of the intensity areas in the development of new types of electronic and photonic materials^1^,^2^,^3^,^4^,^5^,^6^. In particular, SiC and BN semiconductor nanocrystals have enabled and transformed research in technologically important areas including biolabeling^7^,^8^,^9^, microelectromechanical (MEMS) and optoelectronic devices^10^,^11^,^12^. For example, emission wavelengths can now be precisely controlled by the nanostructure composition, the average grain size and the formation of core-shell structures^4^,^13^,^14^,^15^. Basically, it now is possible to modify exaction confinement for better properties, such as longer lifetime and more strongly emission wavelengths^16^.

Previous studies have shown that the incorporation of SiC heterostructures could improve the emission properties of SiC, which has industrial application^17^. Recently, C–SiC core–shell tetrapods, which exhibit narrow line width photoluminescence spanning the visible to near-infrared range, have been synthesized by sol-gel processing^18^. On the other hand, luminescence property of SiCN is considerably different from that of SiC because of the significant changes in the band gap (SiC bandgap (BG) vs SiCN BG as calculated from G. Y. Sung)^19^,^20^. Therefore, SiCN can be considered as a new type of luminescent semiconductor.

The focus has been paid on the application of SiCN as a composite matrix material in the foregoing studies^8^. Thus, SiCN matrix composites have been made with filler phases of graphite^21^ or carbon nanofibers^22^, while many precursors-derived composites (e.g. SiCN/graphite composite) offer the potentials as semiconductors^22^,^23^. One can propose the potential to form new types of luminescent heterojunction materials through studying various types of precursor derived from SiCN/BN nanocomoposites. In addition to changing precursor structures and compositions, it is also likely that varying the pyrolysis conditions might also provide methods for the optical and electronic properties of the resultant nanomaterials^24^. For electronic and optoelectronic devices, selected construction of a specific nanostructure can be called upon to form interface/heterojunction with controllable properties and the potential to effect channeling or separation of charge carriers.

In spite of multiple and extensive studies on the growth of BN thin films by the ways of chemical vapor deposition (CVD)^7^,^25^,^26^, micro-mechanically assisted cleavage of BN flakes through ultrasonication or solution^27^,^28^,^29^,^30^, we have pursued a simple but efficient thermolysis route to what appear to be quite different materials. In this contribution, the crystalline SiCN−BN core−shell nanocomposites, as well as few-layers BNNSs are obtained via a polyborosilazane pyrolysis route. Large quantities of wafer-like BN nanosheets with 2‒7 atomic layers are also generated, and to some extent have rolled up encapsulating crystalline SiC(N) nanowires, exhibiting superior fluorescence behavior as discussed below.

The motivations for the current studies are focused on exploiting the effect on the heterogeneous composites by changing the composition of nanocomposites to modify their luminescent properties. Meanwhile, we provide a novel approach to fabricate SiC(N)/BN luminescent heterojunction nanocomposites. In principle, by controlling the core-shell nanostructure of the resulting composite may offer innovative properties for photoelectronic sensors and tunneling diode^31^.

In addition, cobalt complexes are conveniently introduced into the precursor polymers to control the composition and catalyze the formation of SiCN/BN nanocmposites^32^,^33^,^34^. Herein, we pyrolysis polyborosilazane precursors containing the facilely synthesized cobalt complex [Co (en)_3_]·Cl_3_ which appears to catalyze the formation of SiC(N) nanowires as well as the contribution to enhance the fluorescence. This method should pave the way for 2D BNNSs loading SiCN/BN heterostructure production and utilization as functional fluorescence for a wide range of biolabeling, photonics^12^,^35^, and optoelectronic devices^9^,^30^.

## Results and Discussion

### Synthesis of SiC(N)/BN nanocomposites and their structural characterization

**Figure 1:**
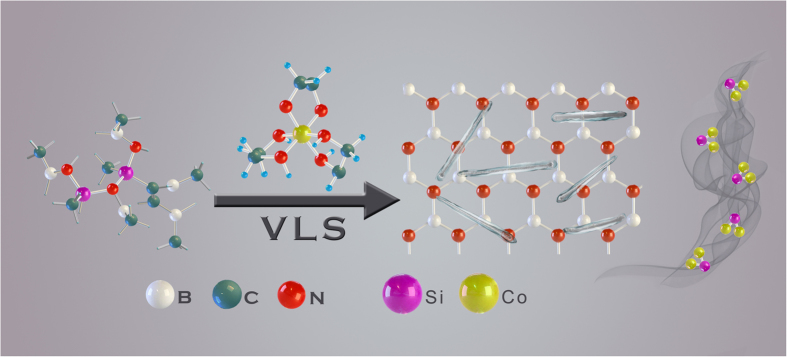
Schematic illustration of the synthesis route of SiCN/BN nanocomposites.

Our synthetic approach to SiC(N)/BN nanocomposites was shown in Fig. 1. In this process, the starting reactants (polyborosilazane and 5 wt% [Co(en)_3_]·Cl_3_ was initially mixed under Ar using planetary ball milling (see experimental section). The resulting powder was transferred into a tube furnace for further thermolysis under Ar at 1600 °C. After cooling to the ambient, lightgrayish powders of millimeter dimensions were obtained.

**Figure 2:**
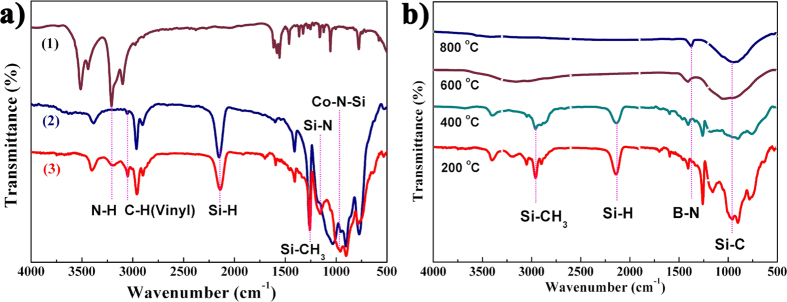
FT-IR spectra of precursor polymers before and after ball-milling are shown in (**a**): (1) [Co(en)_3_]·Cl_3_, (2) PSBN1, (3) cobalt-containing PSBN1; (**b**) FT-IR spectra of the SiC(N)/BN nanocomposites annealed at different temperatures: (navy) 200 °C, (red) 400 °C, (violet) 600 °C (celadon) 800 °C.

During the synthesis, samples were collected at different processes for Fourier transform infrared (FT-IR) spectroscopy measurement and the spectra of the preceramic precursors reacted after cross-linked at 200 °C were shown in Figure 2. According to previous reports^36^,^37^, two reaction pathways occurred during the synthesis of cobalt-containing polyborosilazanes (Co-PSBN1). The first step was the hydrosilylation of the vinyl groups, which gave an explanation to the disappearance of vinyl groups and the decrease in the intensity of Si–H bonds. As the hydrosilation procedure started at 200 °C, almost no change for the intensities of the vinyl and Si–H vibrations were observed in PSBN1^38^. However, the addition of [Co(en)_3_]·Cl_3_ to PSBN1 induced the disappearance of the absorption bands of the alkanes, which demonstrated that the [Co(en)_3_]·Cl_3_ acted as a catalyst for the hydrosilylation. The second reaction route involved the reaction of Si–H bonds of PSBN1 with [Co(en)_3_]·Cl_3_ is expected to the result in the formation of the Si–N–Co linkages^39^. Thus, the synthesis of the precursor cobalt-containing polyborosilazane can be described as illustrated in Fig. 1.

As mentioned above, the one-pot synthesis of Co-PSBN1 was accomplished by the reaction of polyborosilazane (PSBN1) with [Co(en)_3_]·Cl_3_. During the synthesis, samples were collected at different processes for Fourier transform infrared (FT-IR) spectroscopy measurement and the spectra of the preceramic precursors reacted after cross-linked at 200 °C were shown in Fig. 2. FT-IR spectra considered that cobalt complex has acted as an effective component for the formation of SiC in the microstructure of the products. The spectrum of PSBN1, [Co(en)_3_]·Cl_3_ as well as the Co-PSBN1 at 200 °C are shown in Fig. 2a. Upon the addition of [Co(en)_3_]·Cl_3_ to PSBN1, the absorption bands attributed to the C–H stretching at 3050 cm^−1^ and C = C stretching at 1610 cm^−1^ disappeared and relative intensity of the Si–H stretching at 2145 cm^−1^ significantly decreased with respect to the intensity of the Si–CH_3_ bands (Si–C stretching at 780 cm^−1^)^40^. It was noted that new bands are owing to the appeared Co–NSi groups at 970 cm^−1^. The peaks at 2890, 1450 and 870 cm^−1^ were three typical absorption peaks of C–H stretching, N–CH_3_ bending and the characteristic vibrations of Co–N–Si units, respectively, which confirmed that the reaction of [Co(en)_3_]·Cl_3_ with the alkanes groups for the formation of Co–N–Si linkages.

FT-IR spectroscopy of SiC(N)/BN nanocomposite permitted the further characterization of the as-synthesized BN and SiC(N). Figure 2b showed the structural evolution during the annealing of the Co-PSBN1 at different temperatures, in which the lattice vibration modes are caused by the stretching among the silicon, carbon, nitrogen and boron atoms. Strong bands centered at 1375 and 815 cm^−1^ can be assigned to in-plane ring vibration (E_1u_ mode) of the BNNSs and B−N the in-plane stretching vibration, respectively^41^. Moreover, the SiCN nanoparticles were also evidenced by the strong absorption bands centered at 902 and 1160 cm^−1^ corresponded to Si−C and Si−N, respectively. Nevertheless, bands of N–CH_3_ decrease significantly after the pyrolysis above 400 °C and disappeared at temperatures above 600 °C, due to the further reaction between the N–CH_3_ groups of [Co(en)_3_]·Cl_3_ and Si–H groups of PSBN1. Peaks for Si–H vibration at 2145 cm^−1^ and 960 cm^−1^ also decrease due to dehydrocoupling (Si–H/Si–H) in spectra of the pyrolysis temperature at 400 °C. Furthermore, there still existed the characteristic absorption of Co–N–Si groups at 910 cm^−1^ close to the Si−N bands at the pyrolysis temperature up to 600 °C. The obvious decreased intensities of the Si–CH_3_ bands at 2940 and 1255 cm^−1^, as well as Si–CH_2_–Si bands at 2890 and 1290 cm^−1^ were owing to the decomposition of the organic groups.

**Figure 3:**
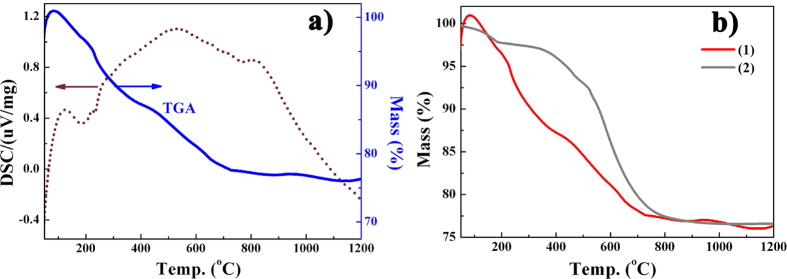
Thermograveimetric analyses of cross-linked Co-PSBN1 and PSBN1 precursors under an argon atmosphere at a scanning rate of 10 Kmin^−1^, (**a**) TGA (blue) -DSC (brown) curves; (**b**) TGA curves of Co-PSBN1 and PSBN1, (1) Co-PSBN1, (2) PSBN1.

The thermal conversion of the Co-PSBN1 precursor into amorphous SiCN/BN composites was investigated by thermogravimetry analysis (TGA) coupled with evolved differential thermal analyzer (DSC) as shown in Fig. 3. The TGA-DSC curves revealed that the polymer-to-ceramic conversion occurred in three steps. At temperatures beyond 800 °C, no mass was observed and the process of ceramization was considered to be completed up to this temperature, leading to a ceramic yield of *ca*. 76 wt%. Cross-linking of the Co-PSBN1 precursor was a combination of vinyl-terminated polymerization and hydrosilylation between vinyl-Si and Si–H groups^42^,^43^. The mass loss was hardly observed in these processes and it contributed to the increase of the ceramic yield of the Co-PSBN1, as compared to the Co-free polyborosilazane. Furthermore, transamination processed between Si–N/Si–N groups and Si–N/Si–N–Co groups occurred with the release of amine fragments, which were verified by the shift of endothermic peaks to the lower temperature on DSC curves. Thus, in the temperature range of 800 °C to 300 °C, the decomposition step is mainly caused by the evolution of Si–CH_3_, Si–H and ethylenediamine fragments. A small amount of weight loss was observed and the dehydrocoupling of Si–H/Si–H or Si–H/–NH– groups were detected (Fig. 3a) at temperature below 230 °C^44^. In addition, transamination processes between Co–NH and Si–N and the decomposition of methane and amido fragments occurred during the polymerization to form the Si–N–Co linkage. Although the reaction occurred, the trace of the introduced cobalt (III) complex has slight influence on the ceramic yield. In addition, transamination processes between Co–NH and Si–N and the decomposition of methane and amido fragments occurred during the polymerization to form the Si–N–Co linkage.

**Figure 4:**
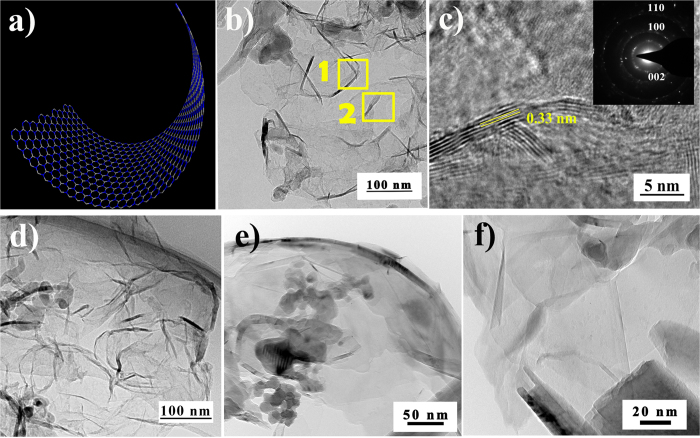
A schematic of the molecular structure of the 2D nanocomposite and transmission electron micrographs of BN nanosheets, which was drawn by Nanotube Modeler. (**a**) Schematic representation of the monolayer BN nanosheet. (**b**) and (**d–f**) The bright field images of *h*-BN nanosheets. (**c**) The HRTEM image of the BNNSs of the region **1** in (**b**), and the inset is the corresponding selected electron diffraction pattern.

After pyrolysis at 1600 °C for 2 h, the precursor (cobalt-containing) polyborosilazanes were transformed into ceramics. We first discuss the characterization of the BN nanosheets of various components in the nanocomposites in this section. Figure 4 introduced the TEM images of high quality BNNSs, which were synthesized during the process of pyrolysis. The EDX pattern of area 2 in Fig. 4b shown in Figure S1 in the ESI (Electronic Supplementary Information) confirmed that the composition of these nanosheets is BN (not graphene). In addition, Fig. 4 recorded the distance between BNNSs revealing well-formed *h*-BN nanosheets. The average thickness of the first- and second-region in Fig. 4b was 3 nm and 4 nm, respectively. The hexagonal atomic structures were obviously determined via the simulation from inverse fast Fourier transform (FFT) of the BNNSs edge segments (Fig. 4c). Each bright spot in the image was for a boron-nitrogen ring (inset of Fig. 4c). The most thickness of BNNSs was less than 4 nm, so the corresponding number of BN layers was less than 10.

**Figure 5:**
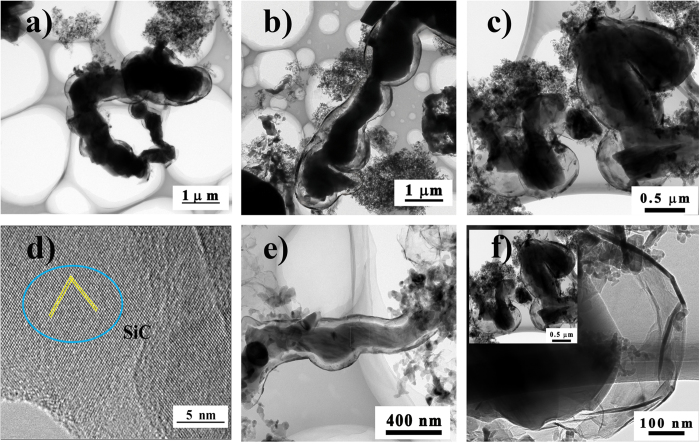
TEM images of SiCN/BN heterostructure. (**a**) A typical view of a single SiCN−BN core‒shell nanostructure. (**d**) HRTEM images of SiC core. (**b**,**c**) and (**e**) The bright field images of SiC(N) nanowires wrapped by BN multilayers at different position, respectively. (**f**) Apart of bright field images of (**c**). Inset showed bright field images of SiCN–BN core–shell nanostructures in distinct amplifications.

Meanwhile, on increasing the initial polyborosilazane content, both fully encapsulated and uniform few-layers BNNSs were concomitantly formed with the increases in both size and density for multilayer BN sheets, per Fig. 4. We also examined BNNSs fragments bound to the metal surface as a primary point for BN nucleation. The formation of nucleation was energetically preferable due to the absence of nanocompensated bonds between boron and nitrogen atoms that is likely the formation of graphene. A possible growth mechanism for the multilayer BNNSs may be the island-on-layer growth model^45^. Interestingly, part of BNNSs appeared to be inclosing contact with the SiC to form the SiCN−BN core−shell nanostructure (Fig. 5).

The SiCN/BN heterostructures consisted of *h*-BN wafers with an average of 5‒25 layers (Fig. 5) and the SiCN−BN core−shell heterostructures with diameters of 50‒400 nm. The SiCN nanowires were prepared by annealing precursor Co-PSBN1 at 1600 °C under Ar. Additionally, most of the encapsulation of SiCN by *h*-BN gave rise to the formation of SiCN−BN core−shell heterostructures during the two-stage nucleation process. SiCN nanowires were prepared by annealing precursor Co-PSBN1 at 1600 °C under Ar.

Take into account these analyses, we suggested that the formation of BN shells was arisen as a classical lattice phenomenon with crystal superlattice theory^46^. By controlling the incorporation of different monomer species into the edges of a 2D sheet, the expected heterostructures can be constructed in a layer-by-layer mode^46^. Consequently, a large class of 2D graphene-like structure was formed in the presence of cobalt as a catalyst. The adatoms were diffused along the BN edges before they were finally incorporated and became part of the growing crystal to form the thin film^47^. Meanwhile, BN species deposited on the surface of SiC nanowires may decrease the energy barrier for the nucleation of BNNSs and the surface energy of SiC nanowires. Moreover, the formation of SiC(N) nanowires was catalyzed through the cobalt nanoparticles and it could be described by the vapor-liquid-solid (VLS) growth mechanism^4^,^47^.

The *h*-BN crystals grew on the surface of catalytically grown SiC(N) nanowires under the effect of CoSi_2_. Subsequently, SiC(N) became encapsulated in *h*-BN at higher *h*-BN local concentrations^6^. This brings about homogeneous core−shell structures and formed SiCN phase. However, the remaining small amount of SiC nanoparticle grows attached to BNNSs, forming the 2D naonocomposites, as shown in Fig. 5a. The high resolution image (Fig. 5d) illustrates that the SiC nanowires in various areas are crystalline phase clearly seen lattice fringes, and the interplanar spacing is about 0.25 nm.

**Figure 6:**
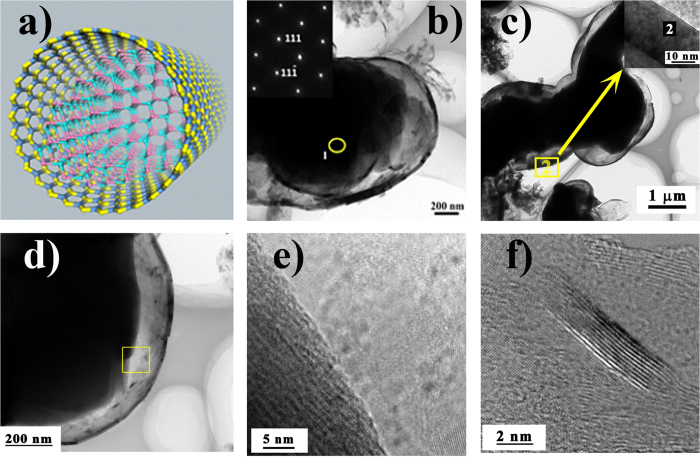
(**a**) The schematic diagram of SiC(N)−BN core−shell nanocomposites. (**b**) and (**c**) Bright field images of SiCN–BN core–shell at different position. The inset of (**b**) and (**c**) showed the FFT pattern for the nanowires HRTEM images of area 2, respectively. (**d–f**) TEM images of the interface of SiCN and BN are shown at (**d**) bright field images. (**e**) and (**f**) HRTEM images of the edges of folding BNNSs. This indicates a 5‒25 nm ranges of BNNSs thickness at different position.

Figure 6a showed the schematic diagram of (SiC)−BN core−shell nanostructure which might be helpful to understand. HRTEM images of the (SiC)−BN core−shell magnified in Fig. 6e was collected with aberration corrected condenser (resolution 1 Å). Fig. 6b introduced the TEM images of SiCN/BN heterostructure, and the inset was the FFT pattern for the area 1 that corresponds to the interspacing of 0.25 nm of HRTEM results. A higher magnification TEM image in Fig. 6d showed that a typical SiC−BN interface strongly suggested a heterojunction structure. Additionally, the most layers of BN shells appeared to be less than 60 in Fig. 6c. This is obviously thicker than the isolated BN nanosheets. Specifically, Fig. 6c depicts BN shell edges of ~25 nm of thicknesses, while BN shell edges observed by HRTEM in Fig. 6f were only 7−9 layers (<3 nm) thickness. The high-resolution image (Fig. 6f) illustrated that the BN nanoshell had an interplanar spacing of 0.33 nm, which was as thin as graphene-like BN. Similarly to the reported^48^, the thickness of BN layers was increasing with the pyrolysis temperature increased in the range of 1400°~1600 °C. The SiCN−BN core−shell nanostructure was not observed when the pyrolysis temperature below 1400 °C^48^. Moreover, the core-shell nanostrcutures was tailored by introducing Co into PSBN1 as a catalyst for the formation of SiCN−BN core−shell. Nevertheless, the core−shell nanostructure could not be found by a similar synthetic route in the absence of Co complex^49^. In addition to BNNSs and BN nanoshells, some BNNSs are formed around the cobalt silicide nanocrystals. The positions of BN nanoshells and BNNSs are around CoSi_2_. This confirmed that cobalt can promote the formation of BN nanowafers. The incorporation of Co catalyzed the *in situ* formation of SiC(N) nanowires, however, that has little contribution to the growth of BN nanowafers.

**Figure 7:**
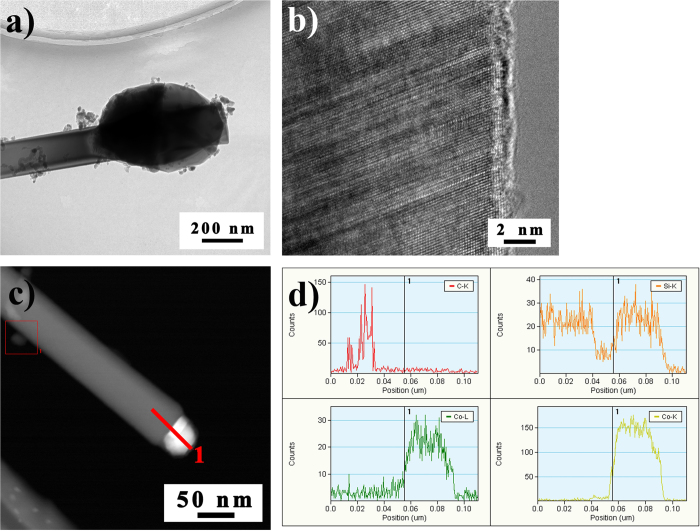
TEM images of SiCN nanowires growing on the metal surface. (**a**) Bright and (**b**) The corresponding HRTEM images of SiC nanowires. (**c**) Dark field images of the SiC nanowires, (**d**) EDS lines-scan profiles of Si, C and Co elements through SiC nanowire/CoSi_2_ heterojunction structure along the line **1** in (**c**).

Figure 7 depicted TEM images of SiC nanowires produced by CoSi_2_ catalysis. Figure S2 in ESI presented the EDX pattern of the top of SiCN nanowires. It is common knowledge that different growth modes correspond to the different contact modes. There is a typical contact mode between CoSi_2_ and the SiC nanowires as observed in Fig. 7a and c. The SiCN nanowire growth processes presented the catalyst at the top, which determined the contact mode in Fig. 7a. It has been suggested that the contact mode depends on the strength of the binding force on the catalyst at the beginning of the SiCN nanowires growth.

**Figure 8:**
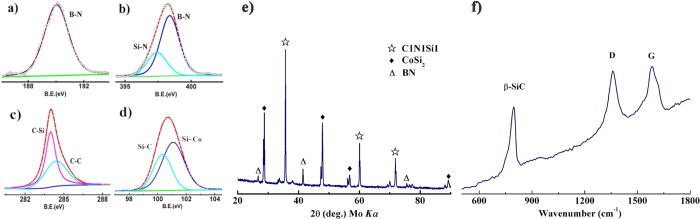
Al *Kα* excited XPS normal emission spectra of boron (**a**), nitrogen (**b**), carbon (**c**), and silicon 1s (**d**) core levels from the SiC(N)/BN nanocomposites, respectively. Intensities of different core levels are too tantamount to scale with arbitrary offsets. (**e**) XRD patterns of SiC(N)/BN nanocomposite. (**f**) Raman spectra of the SiCN/BN nanocomposite after the thermolysis process under Ar atmosphere at 1600 °C.

The lamellar structure of *h*-BN and the (SiC(N)−BN core−shell nanocomposites are also confirmed by XRD, XPS and EDS analyses (see Figure S2 and S1). The crystalline nature of the SiC(N)/BN nanocomposite was established using XRD in Fig. 8e. The sample pyrolysis at 1600 °C in Ar resulted in SiCN, *h*-BN (JCPDS #34-0421) and CoSi_2_ JCPDS #74-1371). The interlayer spacing estimated according to Bragg’s law was 0.33 nm, consistent with the TEM results for BNNSs (Fig. 4c). The peaks at 26.8°, 44.0° and 75.9° are ascribed to (002), (101), (110) planes of *h*-BN, respectively. It directly verified the reflections of B bonding in this experiment, which implies the precipitation of graphite-like boron nitride.

The broad diffraction peaks at 20.0°, 35.6° 60.1° and 71.9° were likely originated from nanocrystal SiCN (JCPDS #074-2309) and graphite carbon. The crystallization of precursor derived SiCN material firstly forms amorphous SiCN, which on further heating (Ar) to 1600 °C forms either *β*-SiC crystallites or a mixed crystalline phase with *β*-SiC and Si_3_N_4_^43^. The mainly crystalline phase is *β*-SiC, because the decomposition of Si_3_N_4_ at 1550 °C results in the content decrease, as shown in equation 1. Sharp peaks in crystalline CoSi_2_ annealed at 1600 °C which are observed at 29.0°, 37.9°, 57.5° and 89.1° 2*θ*^50^. The formation of CoSi_2_ at 1600 °C in Ar atmosphere is likely drove by the known affinity between silicon and cobalt as seen in studies on solid-state joining Si-containing ceramics to cobalt and alloys. As previously reported^51^, the [Co(en)_3_]·Cl_3_ reacts with SiCN phase and gave rise to the formation of an eutectic liquid, which reduced the crystallization temperature of the silicon system to promote the formation of SiC crystals. With the increased annealing temperature, amorphous carbon starts arrangement and the content of silicon is increased. According to the XRD patterns, the dominant phase of cobalt in the sample is CoSi_2_ instead of the metastable CoSi at 1600 °C. The formation of the Co-Si compounds can be described as equation (2).


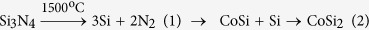


To confirm the presence of BN bonding to the surface of the SiCN nanowires as well as bonding in the formed BN species, the C 1s, Si 2p, B 1s, N 1s and Co 2p core level XPS spectra of the SiC(N)/BN nanocomposites were examined. Figure 8 shows the corresponding XPS spectra providing a mechanism to establish the elemental stoichiometry. In Fig. 8a, the main peak at 190.2 eV corresponds to the B 1s indicating that boron atom is closely associated with nitrogen as anticipated.

As seen in Fig. 8b, the main N 1s peak at 397.6 eV supported a strong B–N bond in BN species that were in good agreement with B 1s spectrum^29^. Both B 1s and N 1s spectra indicate the direction B–N bonding. A shoulder peak at higher energy (398.3 eV) indicated that the formation of the SiCN phase^8^. The C 1s spectrum has two peaks at 284.5 eV and 283.8 eV corresponding to graphite domains and Si−C bonds within SiC(N) in the nanocomposite. Furthermore, the Si 2p spectrum offers peaks at 100.4 eV and 102 eV attributed to Si−C bonds and Si−N bonds^8^,^13^, which are in good agreement with the FT-IR spectra. Attributed to Si−NH−Si groups, the peaks appearing at 3305 and 570 cm^−1^ are N−H stretching and Si−N, respectively^8^. Figure S3 in the ESI indicated that the peak at 778.1 eV can be ascribed to the Co^3+^ 2p_3/2_ state (a Co atom bonding to a Si atom) and 793.7 eV is the specific shake-up peak of Co^3+^ 2p_1/2_ in the Co 2p spectrum. These XPS results imply that the Si−terminated nanocrystals are bonded directly to both N and C atoms. Some of the BNNSs shells are encapsulated in SiC(N). It also appears that CoSi_2_ is generated at high temperature, in accordance with the TEM patterns. Therefore, it will provide support for the SiC nanowires growth through the VLS mechanism in combination of XRD, XPS, and HRTEM (coupled with EDS analysis), but further detailed studies may be needed.

Moreover, Table 1 revealed the chemical composition and possible ceramic formula of PSBN1 and Co-PSBN1 by XPS and ICP-ASE annealed at 800 °C and 1600 °C. The 1/2 atomic ratio of Co/Si was obtained by EDS for Co-PSBN1 annealed at 1600 °C to verify the existence of CoSi_2_, which was consistent with the XRD results. The oxygen content on the surfaces of ceramics (5 < oxygen < 7) was considered the absorption of ceramic powders before measurements. Due to the content of CO_2_ and oxygen was too low that the Si−O bonds and C−O were no appearance in the Si 2p and C 1s spectrum.

Raman spectrum provides useful information about the lattice vibration modes of SiCN/BN nanocomposite. D and G bands are broad after annealing at 1600 °C due to the high disorder of the segregated carbon^48^. Carbon-based materials show strong D and G peaks at 1580 and 1366 cm^−1^, respectively in Fig. 8f as previously reported^52^. These bands are the most striking features of disordered amorphous carbon. Furthermore, overtones at 2700 and 2950 cm^−1^ (2D and D + G modes, respectively) are noted in the spectra. In contrast, the typical peak of BN appears at 1366 cm^−1^, attributed to the E_2g_ interlayer vibration mode of BN, which has also been determined at 1375 cm^−1^ by the FT-IR spectra^53^.

The above results provides evidence to suggest that BNNSs growth in performed SiCN nanowires is related to the synthesis temperature employed^47^. It has been noted that the surfaces of curved SiC nanowires are generally rough with multiple defects, which may serve as nucleation sites for further growth of BN crystals^45^. Moreover, BN has sufficient diffusional mobility at the experimental temperatures such that B and N atoms could be the source of new BN nuclei depositing on nanowire surfaces from the primary nanosheets shells. The incoming BN species adds to the surface of the growing nanosheets, rapidly moving along the surfaces toward the nanosheets edge to covalently bind to the edge atoms. Alternately BN likely diffuses toward the nanowires faster than toward the growing edges due to the weak van der Waals forces. Therefore, BN shells (which wrapped SiC(N) nanowires) tend to grow thicker than the BN nanosheets^54^. Alternately, BN shells grow via the vapor-solid (VS) model, which is similar to the reported^54^,^55^.

In addition, the appearance of CoSi_2_ droplets of SiC nanowires tips was only seen in the presence of Co, suggesting that the growth proceeds via a vapor liquid solid (VLS) mechanism^56^. The melting point of nanoclusters was typically lower than that of the corresponding bulk solids suggesting that CoSi_2_ (Tm = 1480 °C) may form a pseudoliquid state above 1400 °C in this work^57^. According to the Hansen solubility theory, parameters such as catalyst, H−bonding and the cohesive energy density among others are related to the VLS synthesis process. Moreover, various parameters can influence the catalytic activity, for instance, Yang *et al*.^50^ have recently showed the importance of particle topography on the catalytic activity of metal silicide (silicon-rich cobalt silicide) in the growth of SiC nanocrystals. In these studies, the formation of nanowires was proposed to occur via the VLS mechanism of silicon-rich (FeSi_2_) metallic tips. In the current studies, it seems that formation of a Co–Si liquid phase is essential for nucleation and the growth of SiC nanowires, as carbon-solubility is greatly improved at these temperatures^21^.

### Photoluminescence

**Figure 9:**
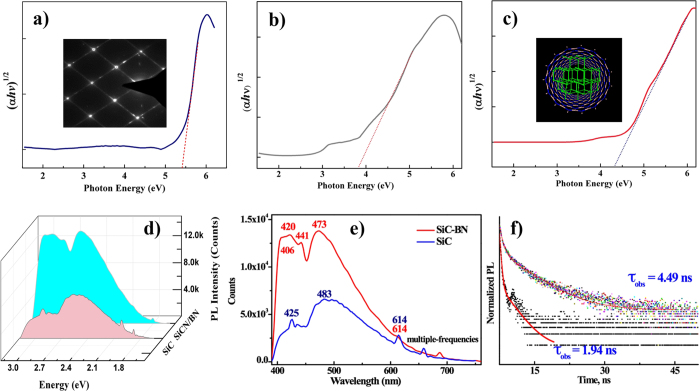
Photonic properties of SiCN/BN nanocomposites. (**a–c**) The UV-Vis diffusing reflectance spectra of BN, SiC and SiCN/BN nanoparticles are shown, respectively. The inset of (**a**) is the FFT pattern for the SiCN nanowires; the inset of (**c**) is the simulation diagram of SiCN−BN core–shell heterostructure. The optical bandgap is estimated around 4.4 eV through the linear fitting in the bottom panel. (**d**) and (**e**) The emission from SiCN/BN nanocomposite and SiC nanoparticles on excitation at 380 nm, respectively. (**f**) A micrograph showing fluorescence lifetime measurements for SiCN/BN nanocomposite and SiC nanoparticle. A variation in lifetime between 1.94 (black) and 4.49 ns (colorized) is attributed toSiC and SiCN/BN nanocomposite, respectively. It is possibly arising from the morphological differences between the SiC(N)−BN core−shell structures and the SiC nanoparticle.

To investigate the fluorescence features, the UV-Vis diffuse reflectance spectra of samples were performed, (Fig. 9). Without any thermal processing, the nanocomposites display a fluorescence performance at room temperature. For comparative purposes, we also characterized SiC nanoparticles without BN shells to compare PL emissions, while typically BN is just an inert, wide band gap semiconductor (*h*-BN, 5~6.4 eV)^7^. Luminescence data was analyzed using the Kubelka-Munk formula, α(*hv*) = A(*hv*-Eg)^n^, where A is the normalization factor, absorbance is the result of the conversion from F(R) = (1-R)^2^/2 R and A = log(1/R), for indirect band gap semi-conductor, n = 1/2. ‘(αh*v*)^1/2^ vs. h*v*’ plot is applicable to indirect gap excitation accordance with semiconductor energy band theory. The effect of the semiconductor heterojunction makes the photon energy of SiC(N)/BN nanocomposite (4.45 eV) between the band gap of SiC nanoparticles (3.8 eV) and *h*-BN (5.46 eV)^24^. Earlier broadband emission of the BN was reported as structured 3.88 eV. Whereas broad 3.10 eV emissions are thought to arising from nanoscale effect as well as nanostructured *h*-BN that attributed to natural impurity point defected by Museur^58^. Wu *et al*. also observed BN nanotubes and BNNSs with broad PL emission bands at 4.2 and 6.05 eV, respectively^9^,^59^. Further, PL emission at 5.46 eV has been attributed to the radiative recombination of donor-acceptor pair transition. Accordingly, the small amount doped CoSi_2_ semiconductor (less than 4 wt% according to XPS and ICP-ASE analysis) into SiC(N) will not affect the bandgap semiconductor substrate.

SiC nanoparticles emission with a characteristic blue-shift interpreted as arising from nanowire stacking quantum effects from isolated SiC(N) nanoparticles, eventhough SiC(N) nanowire dimensions are 50‒400 nm, larger than the Bohr radius (SiC < 3.8 nm)^18^. The characteristic splitting phenomena at the top can also serve as explained by the stacking faults. From the UV-Vis analysis, we believe that this hybrid absorption edge observed in Fig. 9(a–c) represents an impurity state introduced by the BN functionalization to the SiC lattice.

To identify the effect of the semiconductor heterojunctions, photoluminescence (PL) behavioral studies were carried out. Both SiCN/BN nanocomposites and SiC nanoparticles were excited at 380 nm wavelength. Fig. 9d depicts the normalized PL spectra of the SiC(N)/BN nanocomposites at a 380 nm excitation wavelength. In Fig. 9, we can noted that there are five intensive peaks centered at 2.0, 2.60, 2.82, 2.95 and 3.10 eV for the [100] polarization. PL emission centered at 480 nm reached a maximum at an excitation wavelength of 470 nm. The heterojunction located at the interface between the SiCN core and the BN shells, together with the quantum confinement, likely arise from spontaneous polarization between the SiC(N) and *h*-BN regions in SiC(N)/BN heterostructures. Such heterostructures are known for the induction of a strong internal electric field^18^. These results suggest that SiC(N)/BN nanocomposites might be excellent candidates for both optoelectronic and electronic applications^9^.

The solid-state fluorescence lifetimes (τ_obs_) were determined from the luminescent decay profiles for SiC(N)/BN nanocomposites at room temperature by fitting with the monoexponential curves, typical decay profiles are shown in Fig. 9f. Relatively shorter lifetime is observed for SiC nanoparticles (τ_obs_ = 1.94 ns), while the relative longer lifetimes observed for SiC(N)/BN nanocompsites (τ_obs_ = 4.49 ns). This may be arisen from different nonradiative decay channels associated with vibration coupling between the two unique nanostructures or the nanoscale geometry offering a complex and intriguing electronic structure.

Reported defect luminescence in SiC(N)/BN nanocomposite is predominantly observed only at cryogenic temperatures; consequently the observed photoluminescence from the wrapped SiC(N) may be rationalized as being from a quantum confined nanoscale core-shell heterojunction formed. Spatially indirect transitions in core-shell nanostructure with a larger 3C region would exhibit longer radiative lifetime.

## Conclusions

In summary, this study shows a convenient method to synthesize the interesting heterogeneous structure of SiC(N)−BN core–shell. A simple pyrolysis route promoted by a Co catalyst induces the formation of BNNSs wrapped around SiC(N) nanowires. Moreover, the high crystallinity and graphene-like BNNSs are also fabricated in this case, which is promoted by the VS growth mechanism. CoSi_2_ as the catalyst has been proven to play a key role in the formation of BNNSs. Although thin BNNSs have been synthesized via the traditional CVD method, thin layers and improved convenience of BNNSs can be produced by the polymer-pyrolysis method. These impacts structural modification and structure activity relationships of SiC(N)/BN nanocomposites especially the fluorescent properties. Importantly, the ambient temperature PL of these SiC(N)/BN heterostructures exhibit a twice stronger emission than that of the SiC(N) nanoparticles at the excitation wavelength of 330 nm, which can be attributed to the heterojunction formed on the interfaces of SiC(N) and BN by the heterogeneous nucleation. The resultant novel SiC(N)/BN heterostructures may offer considerable potential for electronic and optoelectronic devices. Thus, future studies are underway to decouple the scale of SiC nanoparticles and increase the proportion of few-layered BN nanosheets via different controlled chemical processes. It would be also helpful to apply the similar routes for the preparation of SiC(N)/BN nanocomposite, since those silicon-based polymer are of diversified structural morphology. The SiC(N)/BN nanocomposites, many interesting investigations can be envisioned which will lead to important applications in the field of UV light emitting devices for optoelectronic, the dielectric layers for graphene electronic, the ideal of high power laser and detector window materials.

## Methods

### Materials and methods

The photosensitive precursor was synthesized by the functionalization of polyborosilazane (PSBN1) which was the product of polysilazane modified by borane tetrahydrofuran (BH_3_.THF) (Aldrich) in anhydrous tetrahydrofuran-toluene mixed solvent. In a typical process, 12.0 g polysilazane was dissolved in 30 ml of toluene in a 200 ml three-necked Schlenk flask with a magnetic stirrer and a ground joint reflux condenser, then cooled to −10 °C through ice-salt bath (CaCl_2_.6H_2_O, NH_4_Cl and NaNO_3_). 1.5 ml of BH_3_.THF dissolved in 30 ml of tetrahydrofuran and then added dropwise into the solution by syringe, and the mixture was stirred for 3 h at −10 °C. Subsequently, the sample was allowed to reflux for 12 h. After the removal of the solvent in vacuum, the white viscous liquid polyborosilazane was obtained with Si/B ratios of 2/1. This reaction was performed in a purified argon atmosphere *via* the standard Schlenk technology^22^,^52^. In addition, the [Co(en)_3_]·Cl_3_ complex was synthesized by CoCl_2_ (Aldrich) and ethylenediamine (Aldrich), as described previously^60^.

The powders were patched in the ratio of 95 wt% of the polyborosilazane and 5 wt% Co[(en)_3_]·Cl_3_ complex in anhydrous THF under magnetic stirring for 2 h, and a sticky mixture was obtained. The mixture was ball milled with 4 harden steel balls in a stainless steel vessel filled with high purity argon for 12 h (rotating speed: 5000 min^−1^, ball-to-materials weight ratio: 20:1, 8 mm in diameter). Following milling, a subsequent annealing at 1600 °C (heating rate: 2.5 K·min^−1^) for 2 h in Ar at a flow rate of 40 scam as a carrier gas. The mixture was then loaded into an alumina boat which was placed at the center of a tubular furnace. Then, the furnace was cooled naturally to ambient temperature under the protection of Ar flow. After that, a large quantity of lightgray product was obtained in the boat.

### Characterization

The FT-IR spectrum was recorded between 4000 cm^−1^ and 500 cm^−1^ using a Perkin-Elmer Spectrum 100 spectrophotometer. For this measurement, the sample was prepared as a KBr pellet. The electronic structure of samples was analyzed by XPS (Kratos, ULTRA AXIS DLD) with monochrome AlK*α (hv* = 1486.6 eV) radiation. All binding energies were calibrated by referencing to C 1 s (284.6 eV). The element content of polyborosilazanes and SiCN/BN nanocomposites were detected by XPS and ICP-ASE (Agilent ICP-OED 720, Australia) measurement (to characterize Si and B content). The powder X-ray diffraction (PXRD) measurements were taken on a PANalytical Empyrean (Netherlands) a linear position sensitive detector within an angular range of 10° to 90°, using Cu Kα radiation (λ = 0.15406 nm). Microstructure characterization at high magnifications, reciprocal space and compositions analysis was carried out using a field emission transmission electron microscope (TEM, Tecnai G2F30, 300 kV, FEI Company, US). Energy dispersive X-ray spectrum (EDS) and electron energy loss spectrum (EELS) was performed to determine the crystalline structure, atomic arrangements and chemical nature of samples. Thermogravimetric (TGA) and differential scanning calorimetry (DSC) analysis were performed on a simultaneous thermal device (STA, 449 C Jupiter, Netzsch, Germany) with a heating rate of 10 K min^−1^ under argon atmosphere (a gas flow of 50 mL min^−1^) in a temperature range of 40° to 1 300 °C.

UV-Vis diffuse reflectance spectra were recorded on a double beam Perkin-Elmer Lambda 950 spectrometer. The sample was measured in the wavelength range of 200‒800 nm using a 2 mm path-length four sides polished quartz cuvette as the sample holder. For the measurement of PL emission spectra, the PL spectra was measured with a FLSP920 Combined Steady State Fluorescence and Luminescence Lifetime Spectrometer (a pulsed Xenon lamp is equipped as the source of excitation) at room temperature. Luminescence lifetimes were recorded on a single photon counting spectrometer with nanosecond pulse lamp as the excitation. The accuracy of the instrument was ± 1** **nm. The sample was placed in a 10 mm four sides polished quartz cuvette.

## Additional Information

**How to cite this article**: Zhang, Q. *et al*. Facile synthesis, microstructure and photophysical properties of core-shell nanostructured (SiCN)/BN nanocomposites. *Sci. Rep.* **7**, 39866; doi: 10.1038/srep39866 (2017).

**Publisher's note:** Springer Nature remains neutral with regard to jurisdictional claims in published maps and institutional affiliations.

## Supplementary Material

Supplementary Information

## Figures and Tables

**Table 1:** Chemical composition of polyborosilazanes (Co-PSBN1 and PSBN1) and SiCN/BN nanocomposites annealed at different temperatures.

Sample	Annealed temperature	Elemental analysis (wt%)					Empirical formula
		Si	C	N	B	Co	
PSBN1	800 °C	42.92	23.27	28.51	5.29	—	SiC_1.7_N_1.4_B_0.5_
PSBN1	1600 °C	45.67	24.56	23.52	6.25	—	SiC_2.0_N_0.7_B_0.5_
Co-PSBN1	800 °C	43.94	23.56	24.97	5.05	2.48	SiC_1.31_N_1.19_B_0.67_Co_0.03_
Co-PSBN1	1600 °C	50.44	23.59	16.14	6.52	3.31	SiC_1.13_N_0.66_B_0.75_Co_0.03_
