# Supplementary material for: Facile synthesis, microstructure and photophysical properties of core-shell nanostructured (SiCN)/BN nanocomposites
Source: Sci Rep. 2017 Jan 13;7:39866. doi: 10.1038/srep39866 (PMC5233973; doi:10.1038/srep39866)
Supplement: Supplementary Information [file srep39866-s1.doc]

**Supplementary Information**

**Facile synthesis, microstructure and photophysical properties of core-shell nanostructured (SiCN)/BN nanocomposites**

**Qian Zhang^1^, Dechang Jia ^1^,*, Zhihua Yang^1^,*, Delong Cai^1^, Richard M. Laine^2^,** ^+^**, Qian Li^1^ and Yu Zhou^1^**

^1^ Institute for Advanced Ceramics, School of Materials Science and Engineering, Harbin Institute of Technology, Harbin 150001, PR China

^2^ College of Engineering Materials Science and Engineering, University of Michigan, Michigan, United State

- Corresponding. [dcjia@hit.edu.cn](mailto:dcjia@hit.edu.cn); [zhyang@hit.edu.cn](mailto:zhyang@hit.edu.cn)

^+^  The author contributed equally to this work

**Supplementary Information Figure:**

**Characterization**


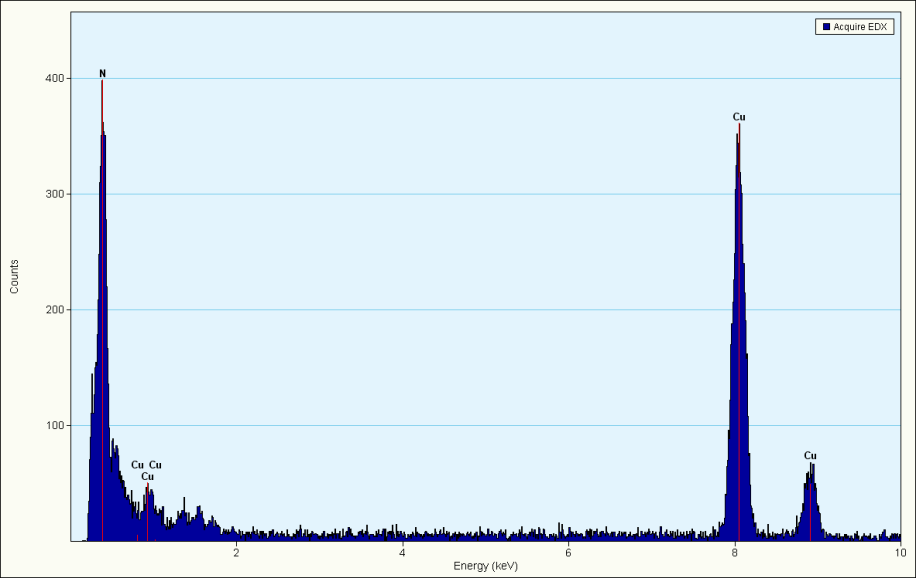


Figure S1 EDX pattern of BN in area **2** of Figure 4. The boron element isn’t included, because of the boron is the light element, which is out of the range of detection. However, there is no carbon element appearence in the pattern.


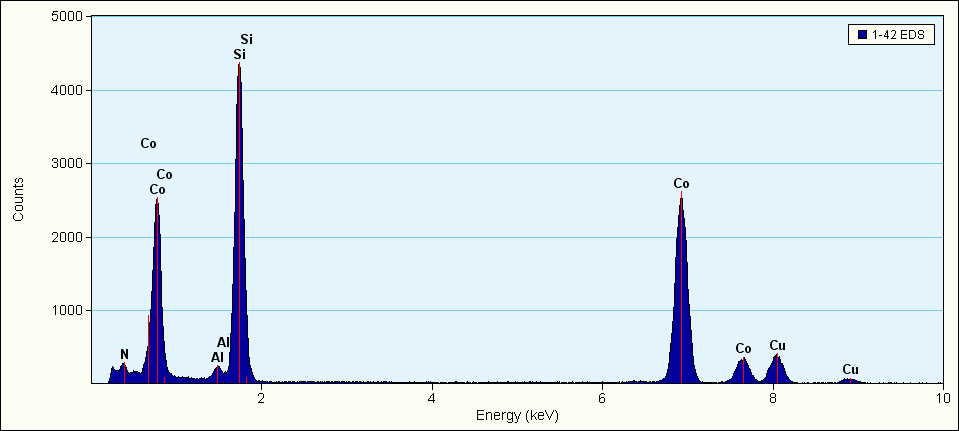


Figure S2 The corresponding EDX pattern of cobalt atoms in area **1** of Figure 5a, which include the SiCN nanowires in Figure 5. The copper element is from the micro grid, and very little aluminum element is introduced by the impurities.


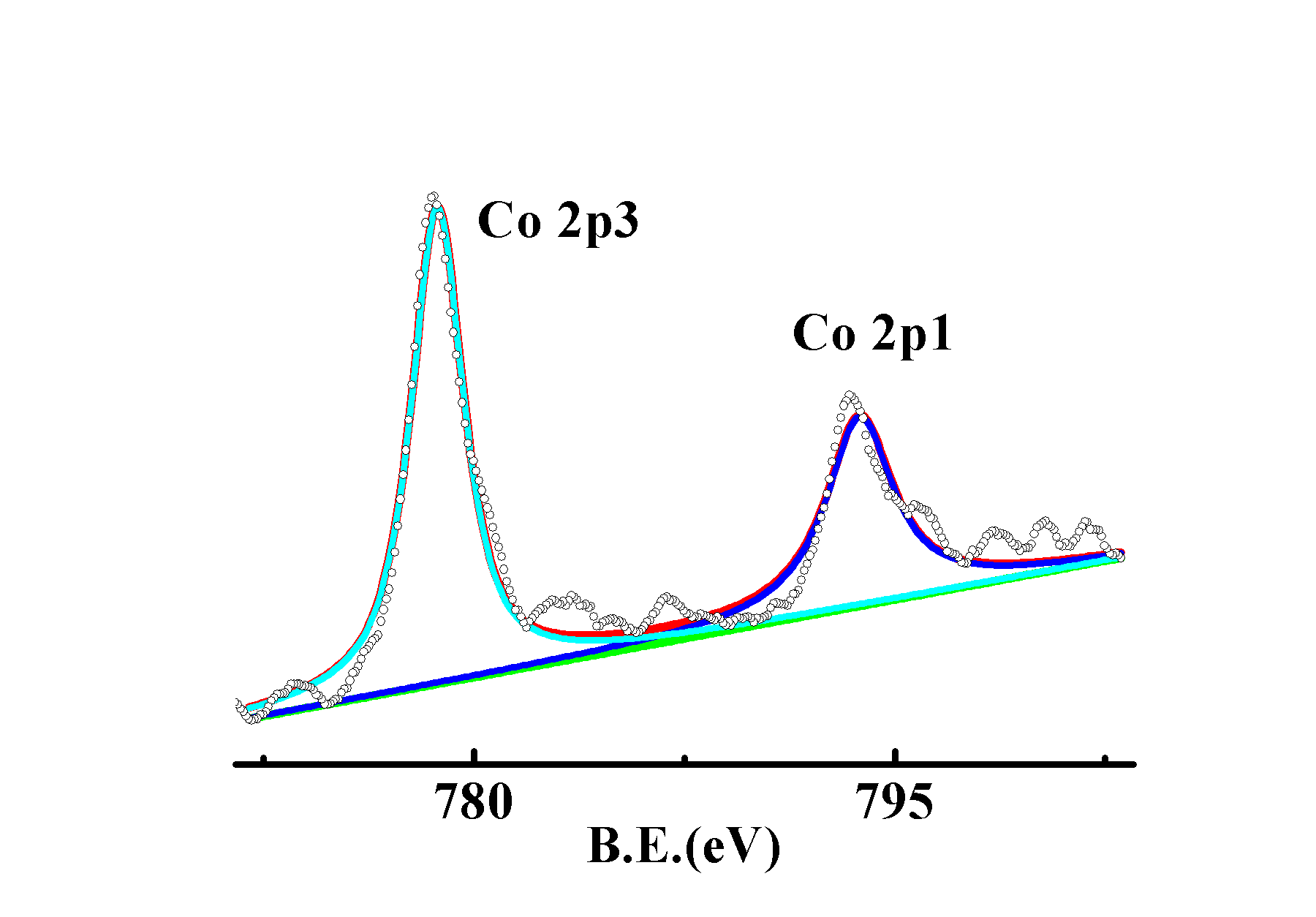


Figure S3 Co 2p XPS spectrum acquired from the SiC(N)/BN nanocomposites powder. It indicated that the peak at 778.1 eV can be ascribed to the Co^3+^ 2p_3/2_ state (a Co atom bonding to a Si atom) and 793.7 eV is the specific shake-up peak of Co^3+^ 2p_1/2_ in the Co 2p spectrum.
